# Geniposide inhibits proliferation and induces apoptosis of diffuse large B-cell lymphoma cells by inactivating the HCP5/miR-27b-3p/MET axis

**DOI:** 10.7150/ijms.51329

**Published:** 2020-09-23

**Authors:** Linjun Hu, Junjun Zhao, Yang Liu, Xin Liu, Qiliang Lu, Zhi Zeng, Lifen Zhu, Xiangmin Tong, Qiuran Xu

**Affiliations:** 1 The Medical College of Qingdao University, Qingdao, Shandong 266071, China; 2 Key Laboratory of Tumor Molecular Diagnosis and Individualized Medicine of Zhejiang Province, Zhejiang Provincial People's Hospital (People's Hospital of Hangzhou Medical College), Hangzhou, Zhejiang 310014, China; 3 Graduate Department, BengBu Medical College, BengBu, Anhui 233030, China

**Keywords:** Geniposide, DLBCL, HCP5, miR-27b-3p, MET, cell proliferation, apoptosis

## Abstract

Diffuse large B-cell lymphoma (DLBCL) is commonly treated with R-CHOP, but ~30 to 50% of the patients are poorly responsive to this strategy. Geniposide, an extract from the *Gardenia jasminoides* Ellis, plays antitumor roles in human gastric cancer, hepatocellular carcinoma, and oral squamous carcinoma. However, the effects of geniposide treatment on DLBCL cells, as well as its underlying mechanism, are still unknown. Here, we found that geniposide inhibited the proliferation of OCI-LY7 and OCI-LY3 cells in a dose-dependent manner. Furthermore, geniposide increased the percentage of apoptotic cells and upregulated the levels of cleaved PARP and cleaved caspase-3 in DLBCL cells. Interestingly, geniposide treatment significantly reduced the expression of the long noncoding RNA HLA complex P5 (lncRNA HCP5) in DLBCL cells. HCP5 expression was revealed to be upregulated in DLBCL tissues and cell lines. Moreover, HCP5 knockdown resulted in proliferation inhibition and apoptosis in OCI-LY7 and OCI-LY3 cells. miR-27b-3p was predicted as a potential target of HCP5 using the lnCAR web tool. Both HCP5 silencing and geniposide treatment increased the level of miR-27b-3p in DLBCL cells. Accordingly, a luciferase reporter assay identified miR-27b-3p as a direct target of HCP5. The expression of miR-27b-3p was upregulated and inversely correlated with the HCP5 level in DLBCL tissues. HCP5 knockdown reduced MET protein expression, which was subsequently rescued by miR-27b-3p silencing in DLBCL cells. Importantly, the restoration of MET partially reversed the geniposide-induced proliferation inhibition and apoptosis of DLBCL cells. In conclusion, geniposide inhibits the proliferation and induces the apoptosis of DLBCL cells at least partially by regulating the HCP5/miR-27b-3p/MET axis, indicating a potential strategy for DLBCL treatment.

## Introduction

Diffuse large B-cell lymphoma (DLBCL) is mainly identified as two subgroups, activated B-cell-like (ABC) and germinal center B-cell-like (GCB)^1^. R-CHOP (rituximab plus cyclophosphamide, doxorubicin, vincristine, and prednisone) has been confirmed to be the standard therapeutic strategy for DLBCL and improves the clinical outcome of patients^2^. Unfortunately, ~ 30% to 40% of DLBCL patients will relapse^3^, ^4^. Thus, it is important to discover novel anti-DLBCL targets and explore underlying molecular mechanisms.

Geniposide, an extract from *Gardenia jasminoides* Ellis, has been recognized as an antitumor reagent in human cancers^5^. For example, geniposide treatment exerts an antiproliferative effect on an oral squamous carcinoma cell line (HSC-3) *in vitro*^6^. Geniposide inhibits the proliferation, migration, and invasion potentials of hepatocellular carcinoma (HCC) cells and induces apoptosis^7^, ^8^. In gastric cancer (GC), geniposide treatment also has inhibitory effects on the malignant biological behaviors of cancer cells^9^. Additionally, several studies have reported that penta-acetyl geniposide plays a suppressive role in the growth, cell cycle progression, apoptosis resistance, and metastasis of C6 glioma cells^10^-^13^. However, the effects of geniposide on DLBCL cells remain unclear.

Long noncoding RNAs (lncRNAs), which are transcripts longer than 200 nucleotides with no or feeble protein-coding potential, account for a large portion of the mammalian transcriptome^14^. Accumulating evidence supports the hypothesis that lncRNAs participate in regulating gene transcription, mRNA stabilization, and protein synthesis and degradation^15^. Furthermore, lncRNAs have been crucial regulators in the occurrence and progression of various human cancers, including DLBCL^16^-^21^. The HLA complex P5 (HCP5) is a cancer-related lncRNA and has been investigated in breast cancer^22^, follicular thyroid carcinoma (FTC)^23^, lung adenocarcinoma (LUAD)^24^, glioma^25^, and GC^26^. HCP5 is frequently overexpressed in glioma tissues and contributes to the proliferation, apoptosis resistance, migration, and invasion of cancer cells by regulating the miR-139/RUNX1 axis^25^. Transcription factor SMAD3-mediated HCP5 facilitates the growth and metastasis of LUAD by targeting the miR-203/snail pathway^24^. Moreover, HCP5 expression is induced in gastric cancer cells by mesenchymal stem cell (MSC) and plays an essential role in promoting chemoresistance and the stemness of tumors^26^. However, the expression, biological function, and related regulatory mechanism of HCP5 in DLBCL are unknown yet. A recent study reports that geniposide exerts antitumor effects by downregulating lncRNA HULC in gastric cancer^9^. Thus, it is worth investigating the regulatory effect of geniposide on HCP5 expression in DLBCL cells.

In this study, the effects of geniposide on cell proliferation, apoptosis, and HCP5 expression in DLBCL cells were determined. Furthermore, the role of HCP5 in DLBCL and its related mechanism was investigated. We found that geniposide repressed cell proliferation and increased apoptosis at least partially by regulating the HCP5/miR-27b-3p/MET axis in DLBCL.

## Material and Methods

### Patients and tissue samples

Forty-eight DLBCL samples and 14 reactive lymph node hyperplasia (RLH) specimens were harvested with informed consent after approval by the Ethics Committee of Zhejiang Provincial People's Hospital. The clinicopathological characteristics of the patients were previously described^16^.

### Cell culture and transfection

Human DLBCL cell lines (OCI-LY7 and OCI-LY3) were previously purchased from the ATCC (Manassas, VA, USA) and maintained in our lab under standard culture conditions as previously mentioned^16^. Normal B-lymphocytes were obtained from a healthy donor as previously described^16^. The cells were treated with geniposide (Sigma-Aldrich, St. Louis, Missouri, USA) at the corresponding concentration.

HCP5 shRNA and nontargeting shRNA (NT shRNA) were provided by GenePharma (Shanghai, China). The miR-27b-3p mimics/inhibitor and negative control (NC) mimics/inhibitor were purchased from RIBOBIO (Guangzhou, China). The pcDNA3.1-MET was generated by inserting the cDNA product of MET into the pcDNA3.1 vector (Invitrogen, Carlsbad, CA, USA). The transfection was performed with Lipofectamine 2000 (Invitrogen) following the manufacturer's protocols.

### Cell proliferation

Cells (4×10^3^ per well) were seeded into 96-well plates. Subsequently, 10 μL Cell Counting Kit-8 (CCK-8, Dojindo Laboratories, Dojindo, Japan) solution was added into each well at the corresponding time points. After incubation for 4 h, the optical density at 450 nm of each well was measured by a microplate reader.

### Apoptosis assay

Cell apoptosis was detected using a PE Annexin V Apoptosis Detection Kit I (#559763, Becton Dickinson Bioscience, San Jose, CA, USA) and the BD FACSCanto™ II Flow Cytometry System (BD, Bioscience, San Jose, CA, USA) as previously described 17.

### Western blotting

Total protein was extracted from the DLBCL cells using RIPA lysis buffer (Beyotime, Shanghai, China), and the protein concentration was measured by a BCA Kit (Pierce, Rockford, IL, USA). The proteins were subjected to 10% SDS-PAGE and transferred onto polyvinylidene fluoride (PVDF) membranes (Millipore, Bedford, MA, USA), which were subsequently blocked with 5% skimmed milk for 2 h. The membranes were then incubated with primary antibodies against cleaved PARP (#5625, CST, Beverly, MA, USA), cleaved caspase-3 (#9694, CST), MET (#8198, CST) and β-actin (sc-8432; Santa-Cruz Biotechnology, Dallas, TX, USA) overnight at 4 °C and subsequently incubated with an HRP-conjugated secondary antibody (Beyotime) at room temperature for 1-2 h. The immunoreactive bands were visualized with ECL reagents (Millipore) and imaged with the Amersham Imager 600 instrument (GE Healthcare Life Sciences, Beijing, China).

### RNA extraction and quantitative real-time PCR (qRT-PCR)

Total RNA from tissues and cells was extracted with TRIzol reagent (Invitrogen). Reverse transcription was conducted by a PrimeScript Reverse Transcriptase Reagent Kit (Takara, Osaka, Japan) and a TaqMan MicroRNA Reverse Transcription Kit (Applied Biosystems, Foster City, CA, USA). The PCR for HCP5 and miR-27b-3p was performed using a SYBR Premix Ex Taq™ II Kit (Takara) in the BIO-RAD CFX96 instrument (Bio-Rad Laboratories, Hercules, CA, USA). The primers were designed and synthesized by the Shanghai Sangon Biotechnology Co., Ltd. (Shanghai, China). U6 and GAPDH were used as the loading controls for miR-27b-3p and HCP5, respectively. The data were calculated by the 2^-△△Ct^ method. The following primers were used: HCP5, forward primer (GACTCTCCTACTGGTGCTTGGT) and reverse primer (CACTGCCTGGTGAGCCTGTT); GAPDH, forward primer (CAAGGTCATCCATGACAACTTTG) and reverse primer (GTCCACCACCCTGTTGCTGTAG); the Bulge-Loop hsa-miR-27b-3p Primer Set (MQPS0000894-1-200) was purchased from Guangzhou RIBOBIO; U6 RT primer (AAAATATGGAACGCTTCACGAATTTG), forward primer (CTCGCTTCGGCAGCACATATACT) and reverse primer (ACGCTTCACGAATTTGCGTGTC).

### Luciferase reporter assay

The potential binding sites between miR-27b-3p and HCP5 were predicted by the starBase web tool (http://starbase.sysu.edu.cn/)^27^, ^28^. The HCP5 fragment was synthesized by PCR amplification with the genomic DNA and inserted into the pGL3 luciferase reporter vector (Promega, Madison, WI, USA). The potential binding sites of miR-27b-3p in the HCP5 fragment underwent site-directed mutagenesis using a QuikChange Site-Directed Mutagenesis Kit (Agilent Technologies, Santa Clara, CA, USA). The pGL3 vectors containing the wild type and mutant type HCP5 fragment were named as WT or MUT HCP5, respectively. The DLBCL cells that were transfected with WT or MUT HCP5 were cotransfected with the NC and miR-27b-3p mimics, respectively. The relative luciferase activity of each sample was calculated using the Dual-Luciferase Assay System (Promega) according to the manufacturer's instructions.

### Statistical analysis

The data were presented as the mean ± SD from at least three independent experiments. The differences among the groups were calculated with one-way ANOVA or Student's t-test. Statistical analyses were performed using GraphPad Prism 8.0 (GraphPad Inc., San Diego, CA, USA). P<0.05 was considered significant.

## Results

### Geniposide suppresses DLBCL cell proliferation but induces apoptosis

**Figure 1:**
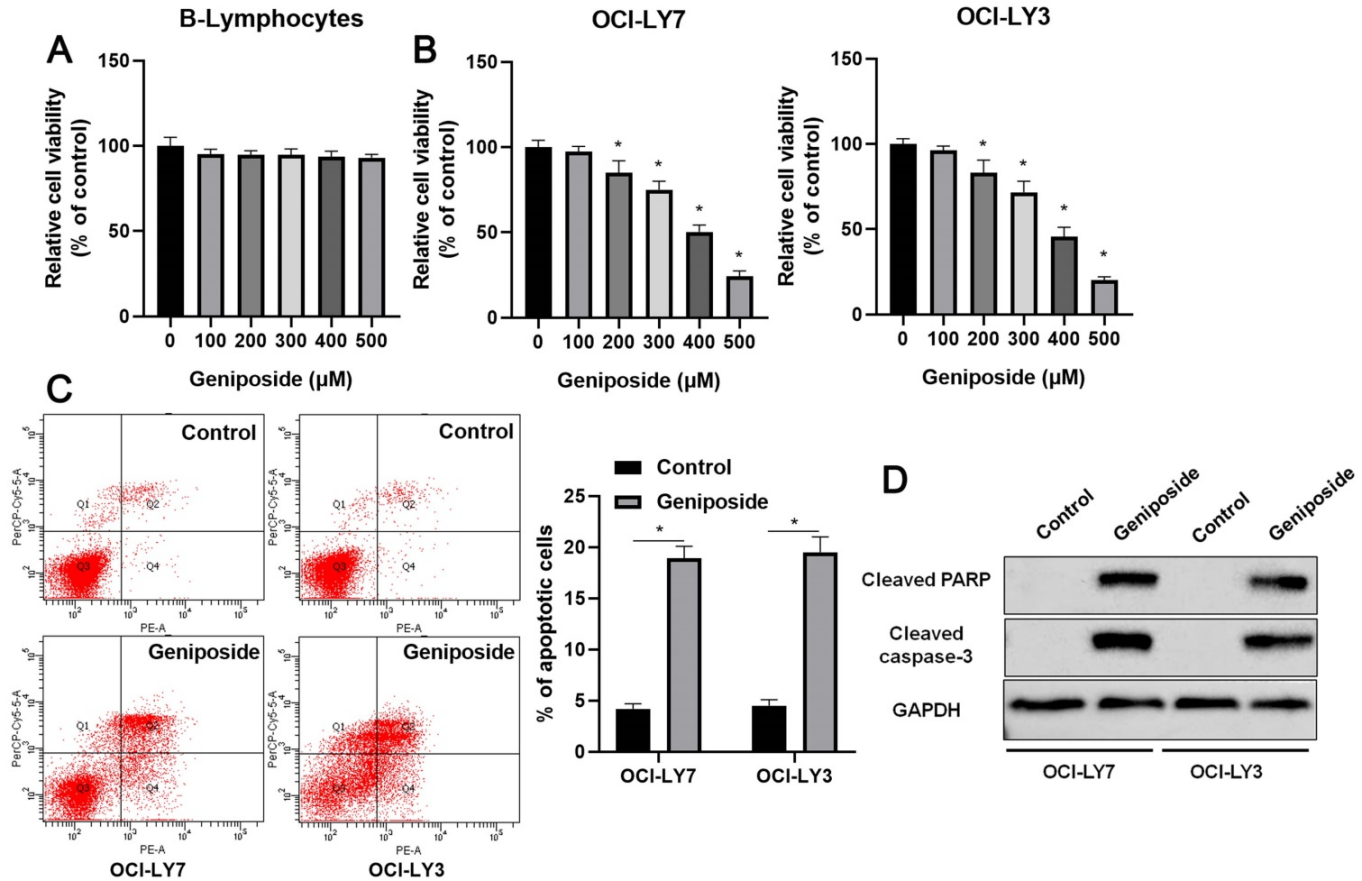
** Geniposide affects proliferation, apoptosis, and HCP5 expression in DLBCL cells**. (A) The viability of B-lymphocytes was detected by a CCK-8 assay after geniposide treatment for 24 h at different concentrations. (B) The viability of OCI-LY7 and OCI-LY3 cells was detected by CCK-8 assay after geniposide treatment for 24 h at different concentrations. (C) Geniposide treatment (500 μM) for 24 h induced the apoptosis of DLBCL cells. (D) Geniposide treatment (500 μM) for 24 h increased the levels of cleaved PARP and cleaved caspase-3 in DLBCL cells. *P<0.05.

First, B-lymphocytes and DLBCL cells were treated with different concentrations (0-500 μM) of geniposide. Geniposide treatment for 24 h at a concentration of up to 500 μM had no cytotoxic effect on the B-lymphocytes (Figure 1A). However, geniposide inhibited the viability of the OCI-LY7 and OCI-LY3 cells in a dose-dependent manner (P<0.05, Figure 1B). Next, geniposide at a concentration of 500 μM was chosen for further studies. We found that geniposide treatment markedly induced the apoptosis of DLBCL cells, as shown by flow cytometric analysis (P<0.05, Figure 1C). Moreover, the Western blotting results indicated that geniposide increased the levels of cleaved PARP and cleaved caspase-3 in both the OCI-LY3 and OCI-LY7 cells (P<0.05, Figure 1D). These data revealed that geniposide exerted an antitumor effect on DLBCL cells.

### HCP5, reduced by geniposide, was highly expressed in DLBCL cells

**Figure 2:**
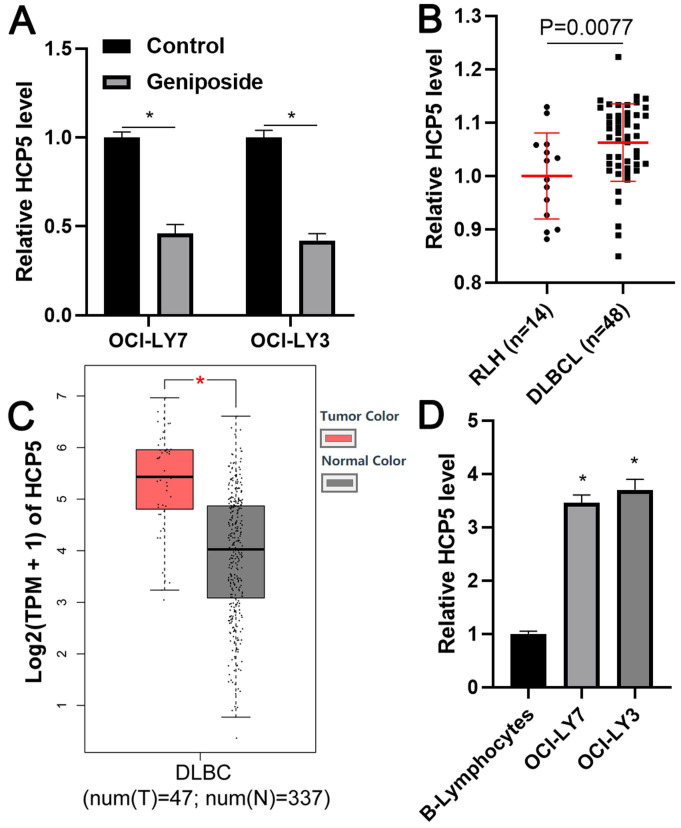
** HCP5 is highly expressed in DLBCL.** (A) Geniposide treatment (500 μM) for 24 h significantly reduced the expression of HCP5 in OCI-LY7 and OCI-LY3 cells. (B) The levels of HCP5 in 48 DLBCL samples and 14 reactive lymph node hyperplasia (RLH) specimens were determined by qRT-PCR. (C) TCGA and GTEx data analysis using GEPIA webtool indicated the upregulated expression of HCP5 in DLBCL. (D) The expression of HCP5 in DLBCL cells was significantly higher than that in B-lymphocytes. *P<0.05.

To explore the regulatory effect of geniposide on HCP5 expression in DLBCL cells, OCI-LY3 and OCI-LY7 cells were treated with geniposide for 24 h and subjected to qRT-PCR to assess HCP5 expression. Interestingly, we found that geniposide treatment prominently decreased the level of HCP5 in DLBCL cells in a dose-dependent manner (P<0.05, Figure 2A and Supplementary Figure 1). The HCP5 expression difference between DLBCL cells and RLH tissues was then determined. We demonstrated that the expression of HCP5 in the DLBCL samples was significantly higher than that in the RLH tissues (P=0.0077, Figure 2B). Furthermore, analysis of the TCGA and GTEx data using GEPIA webtool^29^ consistently revealed the elevated expression of HCP5 in DLBCL tissues compared to normal tissues (P<0.05, Figure 2C). Additionally, the upregulated levels of HCP5 were observed in DLBCL cell lines compared with the B-lymphocytes (P<0.05, Figure 2D). Thus, HCP5 was negatively regulated by geniposide and was frequently overexpressed in DLBCL cells.

### HCP5 knockdown results in proliferation inhibition and apoptosis of DLBCL cells

**Figure 3:**
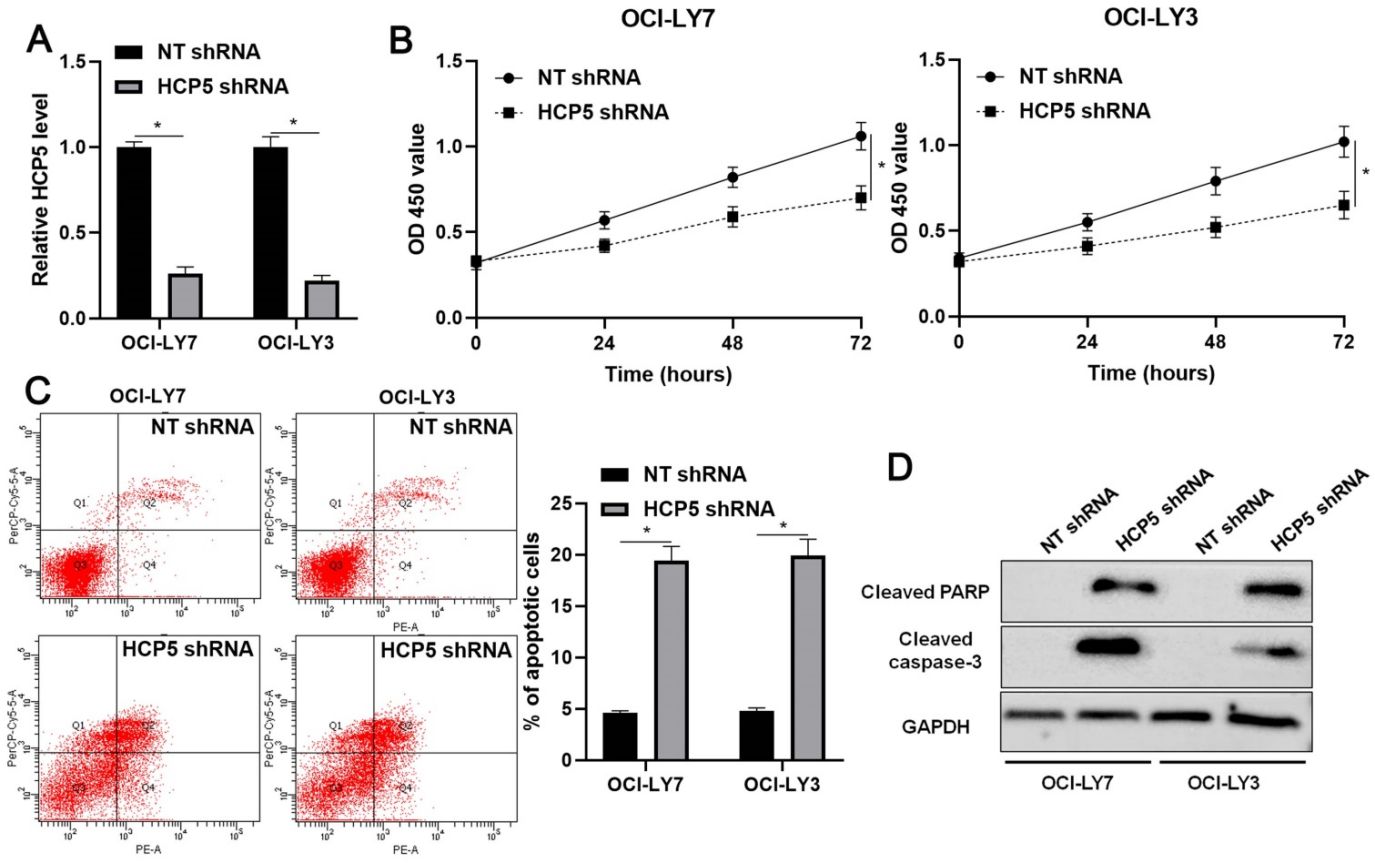
** HCP5 knockdown inhibits proliferation and induces apoptosis of DLBCL cells.** (A) OCI-LY7 and OCI-LY3 cells that were transfected with HCP5 shRNA or nontargeting (NT) shRNA were subjected to qRT-PCR for HCP5 expression. (B) CCK-8 assay indicated that the viability of DLBCL cells was significantly reduced by HCP5 shRNA. (C) HCP5 knockdown prominently induced the percentage of apoptotic DLBCL cells. (D) HCP5 silencing increased the levels of cleaved PARP and cleaved caspase-3 in DLBCL cells. *P<0.05.

Next, the biological role of HCP5 was further confirmed in DLBCL cells. The knockdown of HCP5 was performed in OCI-LY3 and OCI-LY7 cells using a specific shRNA (P<0.05, Figure 3A). The CCK-8 assay indicated that the proliferation of DLBCL cells was significantly reduced by HCP5 knockdown (P<0.05, Figure 3B). Furthermore, the percentage of apoptotic DLBCL cells was markedly increased after HCP5 silencing (P<0.05, Figure 3C). Moreover, HCP5 depletion upregulated the expression of cleaved PARP and cleaved caspase-3 in DLBCL cells (Figure 3D). Collectively, our results showed that HCP5 functions as an oncogene in DLBCL.

### miR-27b-5p is a direct target of HCP5

**Figure 4:**
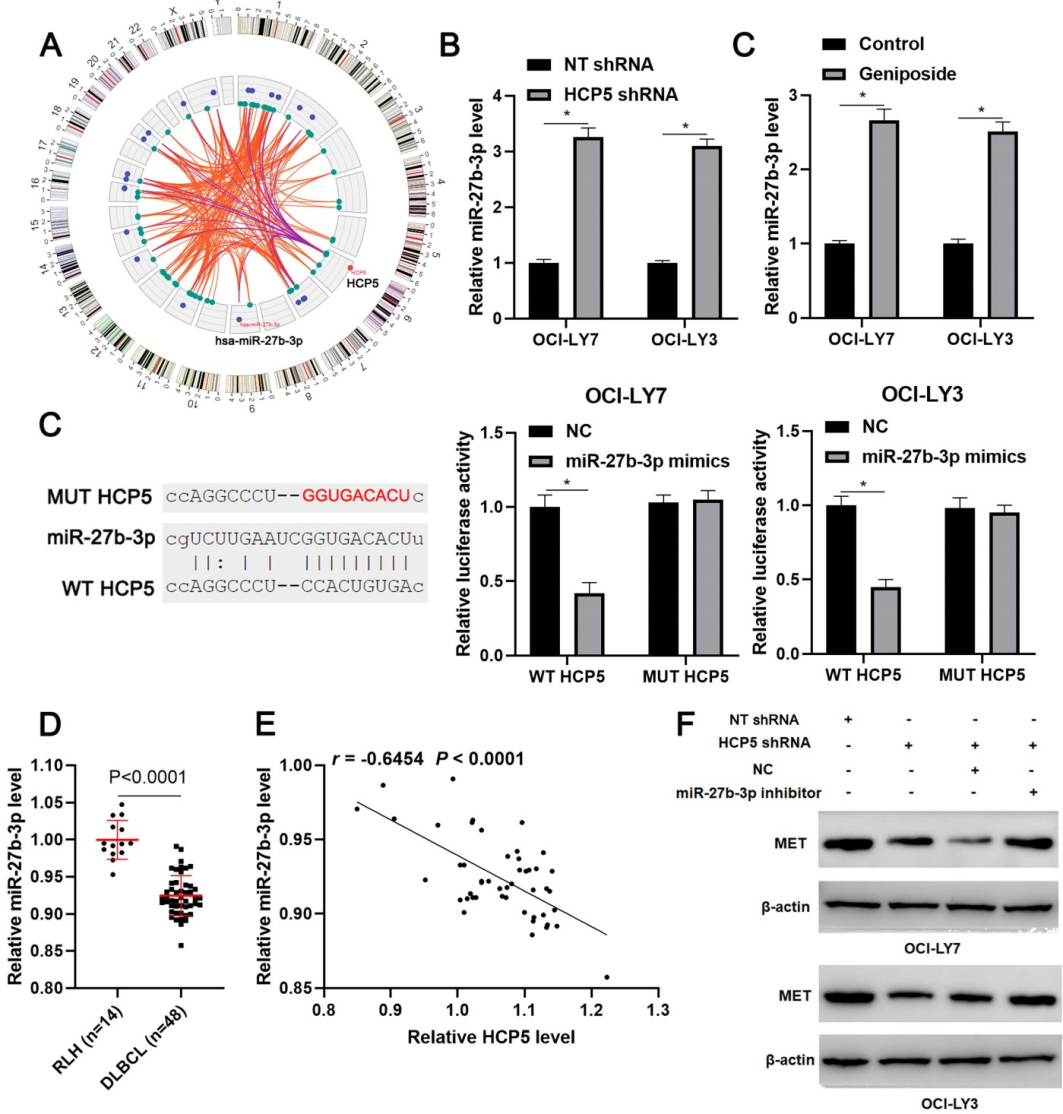
** HCP5 functions as a ceRNA by sponging miR-27-3p**. (A) The ceRNA network of HCP5 was analyzed by the lnCAR webtool. (B) HCP5 knockdown significantly increased the level of miR-27b-3p in DLBCL cells. (C) Geniposide treatment (500 μM) for 24 h prominently upregulated the expression of miR-27b-3p in DLBCL cells. (D) Luciferase reporter vectors containing wild type (WT) or mutant type (MUT) HCP5 and miR-27-3p mimics or negative control (NC) were cotransfected into OCI-LY7 and OCI-LY3 cells, after which the relative luciferase activity was assessed. (E) The levels of miR-27b-3p in 48 DLBCL samples and 14 reactive lymph node hyperplasia (RLH) specimens were determined by qRT-PCR. (F) An inverse correlation between HCP5 and miR-27b-3p expression was observed in DLBCL tissues. (G) OCI-LY7 and OCI-LY3 cells were transfected with corresponding vectors and subjected to western blotting for MET expression. *P<0.05.

Next, we tried to explore the potential mechanism underlying the role of HCP5 in DLBCL. According to the lnCAR (https://lncar.renlab.org/) website^30^, miR-27b-3p, which was previously reported to be a tumor suppressor in DLBCL, was predicted as a candidate target of HCP5 (Figure 4A). We then revealed that HCP5 knockdown significantly increased the level of miR-27b-3p in OCI-LY3 and OCI-LY7 cells (P<0.05, Figure 4B). Notably, geniposide treatment also markedly enhanced the expression of miR-27b-3p in DLBCL cells (P<0.05, Figure 4C). Accordingly, a luciferase reporter assay demonstrated that miR-27b-3p overexpression remarkably reduced the luciferase activity of the plasmid carrying WT HCP5 but not MUT HCP5 (P<0.05, Figure 4D). The miR-27b-3p expression was downregulated in DLBCL tissues compared to RLH tissues (p<0.0001, Figure 4E) and negatively correlated with the HCP5 level (r=-0.6554, P<0.0001, Figure 4F). Moreover, analysis of the TCGA data using starBase webtool^28^ confirmed the negative correlation between HCP5 and miR-27b-3p expression in DLBCL tissues (P<0.05, Supplementary Figure 2). Previous study has demonstrated MET as a direct target of miR-27b-3p in DLBCL^31^. Analysis of the TCGA and GTEx data using GEPIA webtool^29^ demonstrated the upregulated expression of MET mRNA in DLBCL tissues compared to normal tissues (P<0.05, Supplementary Figure 3). Next, we found that HCP5 knockdown reduced the level of MET, which was subsequently rescued by miR-27b-3p silencing in DLBCL cells (Figure 4G). Thus, these results suggested miR-27b-3p/MET axis as downstream targets of HCP5 in DLBCL.

### MET restoration partially reverses the effects of geniposide in DLBCL cells

**Figure 5:**
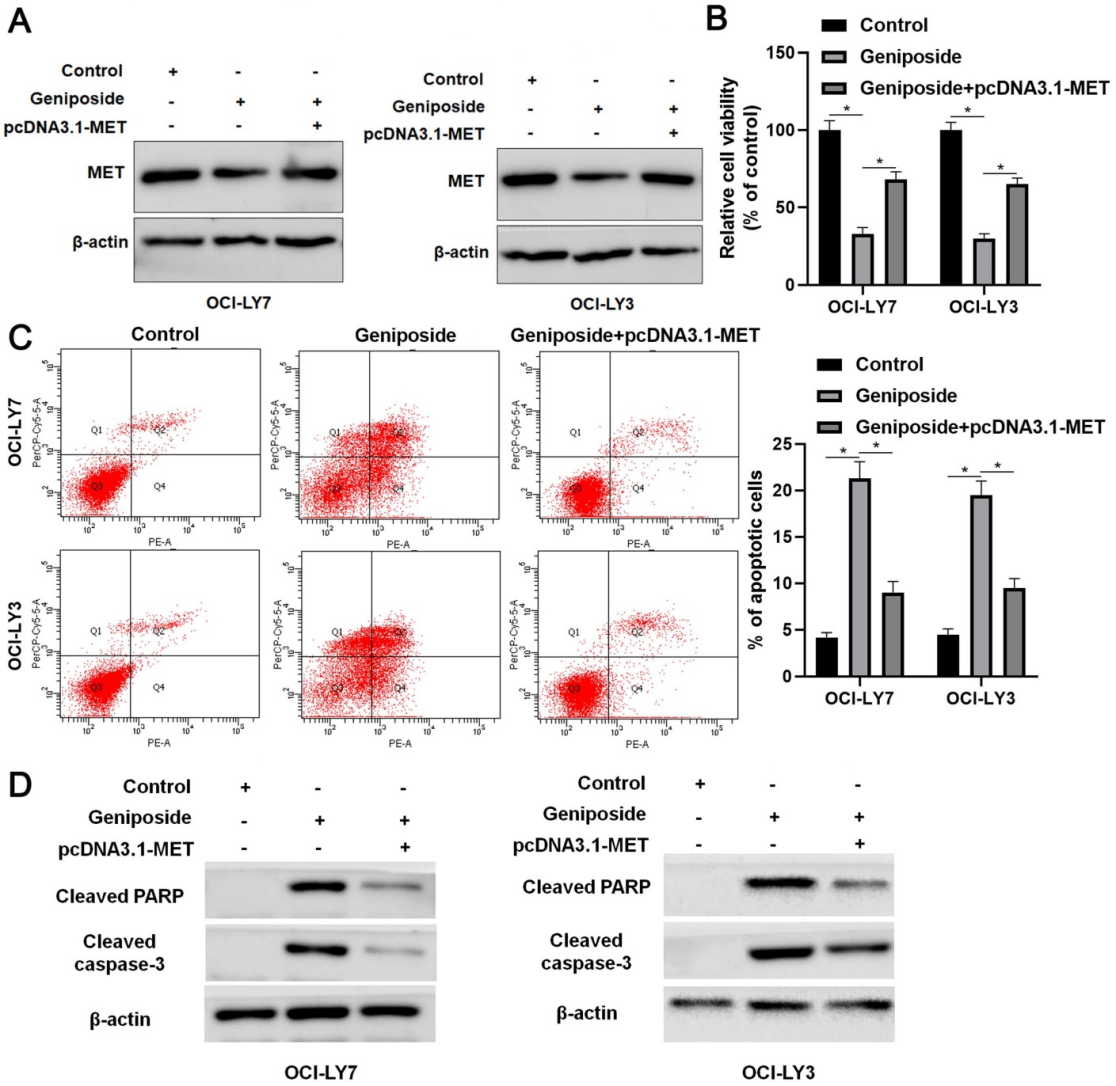
** MET restoration partially abolishes the effects of geniposide on DLBCL cells.** (A) Geniposide (500 μM)-treated OCI-LY7 and OCI-LY3 cells were transfected with pcDNA3.1-MET vector and analyzed by western blotting. (B) The MET restoration increased the viability of DLBCL cells with geniposide treatment. (C) The re-expression of MET reversed the geniposide-induced apoptosis of DLBCL cells. (D) The levels of cleaved PARP and cleaved caspase-3 were increased by geniposide treatment and subsequently reduced by MET restoration in OCI-LY7 and OCI-LY3 cells. *P<0.05.

Rescue experiments were performed to confirm whether the HCP5/miR-27b-3p/MET axis mediated the effects of geniposide in DLBCL cells. Geniposide treatment decreased MET expression, which was restored by transfection of the expression plasmid in OCI-LY3 and OCI-LY7 cells (Figure 5A). The MET restoration significantly enhanced the proliferation of geniposide-treated DLBCL cells (P<0.05, Figure 5B). Furthermore, the re-expression of MET markedly reversed geniposide-induced apoptosis in DLBCL cells (P<0.05, Figure 5C). In addition, the levels of cleaved PARP and cleaved caspase-3 were upregulated by geniposide treatment and subsequently reduced by the MET restoration in DLBCL cells (Figure 5D). Taken together, geniposide exerted antitumor effects at least partially by regulating the HCP5/miR-27b-3p/MET axis in DLBCL.

## Discussion

In the current study, geniposide repressed the proliferation of DLBCL cells and induced apoptosis. Furthermore, we revealed that geniposide treatment resulted in the downregulation of HCP5 and increased the expression of miR-27b-3p in DLBCL cells. HCP5 was highly expressed, while miR-27b-3p expression was reduced in DLBCL tissues compared to RLH tissues. Accordingly, miR-27b-3p was recognized as a direct target of HCP5. Importantly, the miR-27b-3p inhibitor partially reversed the effects of geniposide on DLBCL cell proliferation and apoptosis.

Geniposide is an active extract from the traditional Chinese medicine “Zhizi”. Previous studies have reported that geniposide plays an important role in anti-inflammatory, anti-oxidative stress and antitumor activities 5, 32, 33. For instance, geniposide acts as a protective factor in hypoxia/reperfusion-related brain barrier impairment by reducing inflammation, oxidative stress, and apoptosis *in vitro* 32. Geniposide protects against lipid accumulation by decreasing oxidative stress and inflammation in non-alcohol fatty liver disease (NAFLD) 33. The antitumor effect of geniposide has been indicated in several types of human cancers 6-10. In our study, we first reported that geniposide acted as a tumor-suppressive factor in DLBCL cells by inhibiting cell proliferation and inducing apoptosis. Our data suggested that geniposide might be a potential anti-DLBCL reagent.

The mechanisms underlying the biological roles of geniposide have been widely investigated, and several molecules, as well as signaling pathways, have been found to be regulated by geniposide. For example, geniposide alleviates NAFLD by modulating the Nrf2/AMPK/mTOR signaling pathway 33. The TLR4/MyD88/NF-κB pathway is regulated by geniposide in several human diseases, such as diabetic cognitive impairment 34, acute liver injury 35, and HCC 7. Moreover, miR-21 36, miR-373 37, miR-214 38 and miR-224 8 are under the regulation of geniposide. Recently, several studies have demonstrated the correlation between geniposide treatment and lncRNAs 9, 39, 40. The downregulation of lncRNA THRIL, which is induced by geniposide, protects against hypoxia-induced injury in rat cardiomyocytes 39. Geniposide-induced lncRNA H19 alleviates oxygen and glucose deprivation-mediated injury in PC-12 cells 40. LncRNA HULC is a downstream effector of geniposide in suppressing gastric cancer cell growth, migration, and invasion 9. Here, we found that geniposide treatment induced the downregulation of HCP5 in DLBCL cells. HCP5 is frequently overexpressed in DLBCL tissues, and its knockdown showed similar effects to geniposide on cell proliferation and apoptosis. Further experiments revealed that miR-27b-3p, which was underexpressed in DLBCL, was identified as a direct target of HCP5. miR-27b-3p has been confirmed to be a tumor suppressor, which represses proliferation and enhances apoptosis by targeting MET in DLBCL^31^. Most importantly, our results indicated that HCP5 enhanced MET expression via attenuating miR-27b-3p and MET restoration partially abolished geniposide-induced proliferation inhibition and apoptosis in DLBCL cells. Thus, the HCP5/miR-27b-5p/MET axis might participate in the anti-DLBCL role of geniposide.

In conclusion, our results provide new insight into the tumor-suppressive role of geniposide in DLBCL. Geniposide treatment suppresses proliferation, induces apoptosis, and regulates the HCP5/miR-27b-3p/MET axis in DLBCL cells, which may provide potential therapeutic strategies.

## Conclusions

This study demonstrated that geniposide affected the proliferation and apoptosis of DLBCL cells. HCP5, which was negatively regulated by geniposide, was highly expressed in DLBCL, and promoted cell proliferation and apoptosis resistance. miR-27b-3p was recognized as a direct target of HCP5. HCP5 promoted MET expression by attenuating miR-27b-3p in DLBCL cells. MET restoration partially abolished geniposide-induced proliferation inhibition and apoptosis in DLBCL cells. In summary, geniposide exerted a tumor suppressive role in DLBCL at least partially by regulating the HCP5/miR-27b-3p/MET axis.

## Supplementary Material

Supplementary figures.

## Abbreviations

DLBCL: diffuse large B-cell lymphoma
ABC: activated B-cell-like
GCB: germinal center B-cell-like
R-CHOP: rituximab plus cyclophosphamide, doxorubicin, vincristine, and prednisone
HCC: hepatocellular carcinoma
GC: gastric cancer
LncRNA: long noncoding RNA
HCP5: HLA complex P5
FTC: follicular thyroid carcinoma
LUAD: lung adenocarcinoma
MSC: mesenchymal stem cell
