# Supplementary material for: Geniposide inhibits proliferation and induces apoptosis of diffuse large B-cell lymphoma cells by inactivating the HCP5/miR-27b-3p/MET axis
Source: Int J Med Sci. 2020 Sep 23;17(17):2735–43. doi: 10.7150/ijms.51329 (PMC7645330; doi:10.7150/ijms.51329)
Supplement: Supplementary file 1 — Supplementary figures. [file ijmsv17p2735s1.pdf]

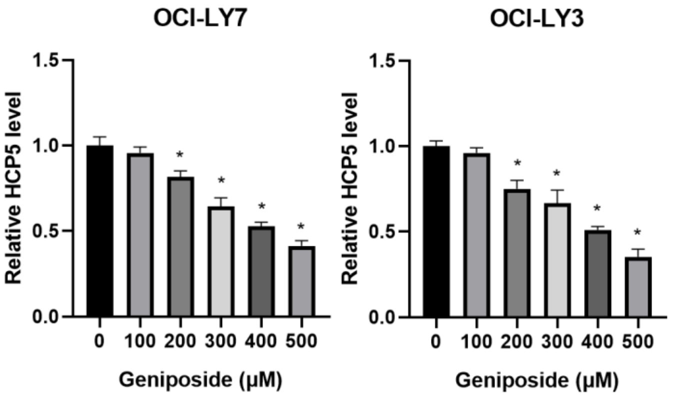

Supplementary Figure 1 Geniposide reduces HCP5 level in DLBCL cells in a dose-dependent manner. The level of HCP5 was detected by qRT-PCR analysis in OCI-LY7 and OCI-LY3 cells after geniposide treatment for 24 h at different concentrations. *P<0.05

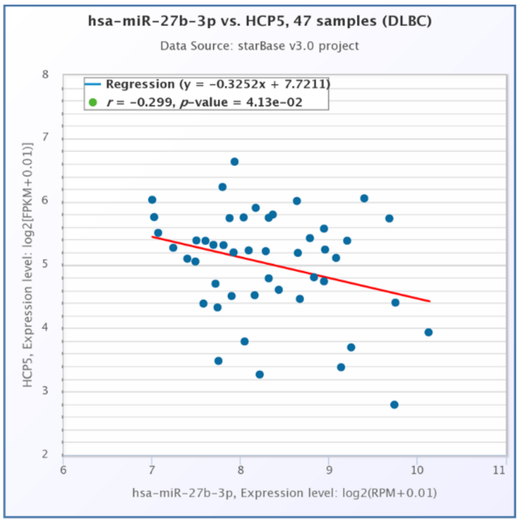

Supplementary Figure 2 The correlation between HCP5 and miR-27b-3p expression in DLBCL tissues. Analysis of the TCGA data using starBase webtool confirmed the negative correlation between HCP5 and miR-27b-3p expression in DLBCL tissues.

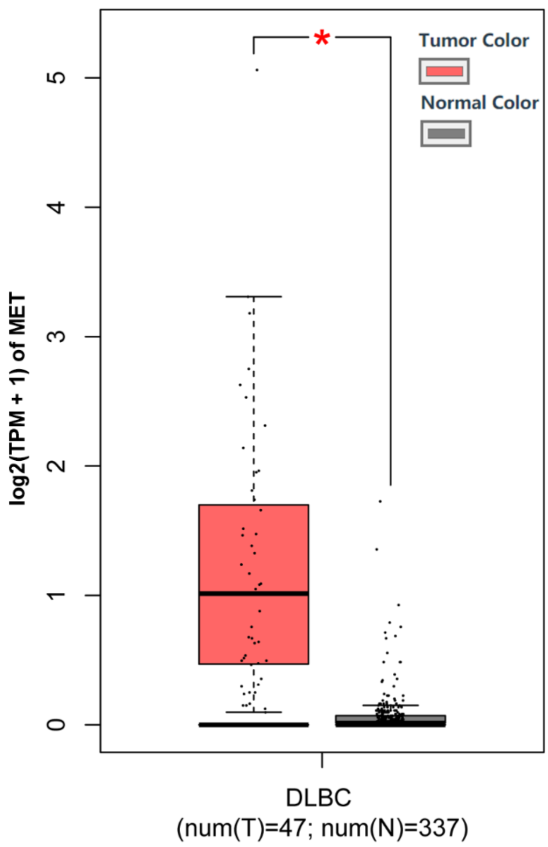

Supplementary Figure 3 The expression of MET mRNA in DLBCL and normal tissues. Analysis of the TCGA and GTEx data using GEPIA webtool demonstrated the upregulated expression of MET mRNA in DLBCL tissues compared to normal tissues. *P<0.05.
